# Genome-wide investigation and functional characterization of the β-ketoadipate pathway in the nitrogen-fixing and root-associated bacterium *Pseudomonas stutzeri *A1501

**DOI:** 10.1186/1471-2180-10-36

**Published:** 2010-02-08

**Authors:** Danhua Li, Yongliang Yan, Shuzhen Ping, Ming Chen, Wei Zhang, Liang Li, Wenna Lin, Lizhao Geng, Wei Liu, Wei Lu, Min Lin

**Affiliations:** 1 College of Biological Sciences, China Agricultural University, Beijing 100094, China; 2 Biotechnology Research Institute, Chinese Academy of Agricultural Sciences, Key Laboratory of Crop Biotechnology, Ministry of Agriculture, Beijing 100081, China; 3 National Centre for Plant Gene Research, Beijing 100101, China

## Abstract

**Background:**

Soil microorganisms are mainly responsible for the complete mineralization of aromatic compounds that usually originate from plant products or environmental pollutants. In many cases, structurally diverse aromatic compounds can be converted to a small number of structurally simpler intermediates, which are metabolized to tricarboxylic acid intermediates via the β-ketoadipate pathway. This strategy provides great metabolic flexibility and contributes to increased adaptation of bacteria to their environment. However, little is known about the evolution and regulation of the β-ketoadipate pathway in root-associated diazotrophs.

**Results:**

In this report, we performed a genome-wide analysis of the benzoate and 4-hydroxybenzoate catabolic pathways of *Pseudomonas stutzeri *A1501, with a focus on the functional characterization of the β-ketoadipate pathway. The *P. stutzeri *A1501 genome contains sets of catabolic genes involved in the peripheral pathways for catabolism of benzoate (*ben*) and 4-hydroxybenzoate (*pob*), and in the catechol (*cat*) and protocatechuate (*pca*) branches of the β-ketoadipate pathway. A particular feature of the catabolic gene organization in A1501 is the absence of the *catR *and *pcaK *genes encoding a LysR family regulator and 4-hydroxybenzoate permease, respectively. Furthermore, the BenR protein functions as a transcriptional activator of the *ben *operon, while transcription from the *catBC *promoter can be activated in response to benzoate. Benzoate degradation is subject to carbon catabolite repression induced by glucose and acetate in A1501. The HPLC analysis of intracellular metabolites indicated that low concentrations of 4-hydroxybenzoate significantly enhance the ability of A1501 to degrade benzoate.

**Conclusions:**

The expression of genes encoding proteins involved in the β-ketoadipate pathway is tightly modulated by both pathway-specific and catabolite repression controls in A1501. This strain provides an ideal model system for further study of the evolution and regulation of aromatic catabolic pathways.

## Background

Aromatic compounds, one of the most abundant classes of natural carbon compounds, accumulate primarily due to the degradation of plant-derived molecules (e.g., lignin). These structurally diverse compounds are independently converted to a small number of structurally simpler common intermediates, such as catechol and protocatechuate, which are subsequently metabolized to tricarboxylic acid intermediates via the β-ketoadipate pathway [1-3]. Therefore, many soil bacteria are characterized by considerable metabolic flexibility and physiological adaptability with a minimum number of functional proteins.

The β-ketoadipate pathway for degradation of aromatic compounds is widely distributed among bacteria. In addition, the microbial degradation of aromatic compounds has tremendous environmental significance. Therefore, the metabolic and genomic characteristics of the aromatic catabolic pathways from *Acinetobacter*, *Pseudomonas*, *Geobacterter *and *Dechloromonas *have been studied extensively [2,4-6]. For example, *A. baylyi *ADP1 (formerly known as *Acinetobacter *sp. ADP1) and *P. putida *KT2440 have long been used as a model for studying aromatic compound biodegradation and have contributed greatly to the elucidation of gene regulation of the β-ketoadipate pathway. In *A. baylyi *ADP1, the β-ketoadipate pathway consists of two parallel branches for the conversion of catechol and protocatechuate, which are derived from benzoate and 4-hydroxybenzoate, respectively [1]. At least 19 genes involved in the peripheral pathways for the catabolism of benzoate (*ben*) and 4-hydroxybenzoate (*pob*) and in the catechol (*cat*) and protocatechuate (*pca*) branches of the β-ketoadipate pathway have been identified in *A. baylyi *ADP1 [4]. *P. putida *KT2440 is another well-characterized bacterium capable of utilizing benzoate and 4-hydroxybenzoate [2,7-9]. Genome sequence analysis of strain KT2440 predicts the existence of the protocatechuate (*pca *genes) and catechol (*cat *genes) branches of the β-ketoadipate pathway [2]. Further enzymatic studies and amino acid sequence data revealed that the *pob*, *pca*, *ben *and *cat *gene products are highly conserved in *Acinetobacter *and *Pseudomonas *strains. These products are usually synthesized in the presence of their respective substrates. Two different regulatory proteins, an XylS-type BenR in *P. putida *[9] and a LysR-type BenM in *A. baylyi *[10], are known to be involved in activating the *ben *gene expression in response to benzoate. In most cases, BenR/BenM is necessary for the *ben *expression but not for the expression of the *cat *genes, which can be regulated by CatR/CatM [11,12]. For example, BenR and CatR jointly activate more than a dozen chromosomal *ben *and *cat *genes responsible for benzoate catabolism in *P. putida *[9,13]. Thus, BenR-CatR or BenM-CatM regulation may serve as a practical model for complex regulatory circuits involved in the biodegradation of benzoate.

Aromatic compounds are not preferred as growth substrates. In most cases, synthesis of the catabolic enzymes is reduced when certain rapidly metabolizable carbon sources are simultaneously present [14]. One such control mechanism is called catabolite repression, which can integrate different signals, thus increasing the complexity of the system [15]. Although the molecular mechanism responsible for global control is not yet well understood, available data suggest that catabolite repression control (Crc) is a component of a signal transduction pathway that modulates carbon metabolism in some soil bacteria. In addition, Crc has also been observed in several *Pseudomonas *species [16]. Very recently, *A. baylyi *Crc was proposed to be involved in determining the transcript stability of the *pca-qui *operon, thereby mediating catabolite repression [17].

The β-ketoadipate pathway is found almost exclusively in soil microorganisms, especially in *Pseudomonas *species, emphasizing the importance of aromatic compound catabolism in this family [18,19]. Establishment of the complete genome sequence of *Pseudomonas *strains enabled mapping of the entire catabolic gene cluster in their chromosomes [2,20,21]. Despite the current extensive knowledge about the aerobic catabolism of aromatic compounds in *Pseudomonas *strains, there remains much more to understand. For instance, the large information gap between sequence information and function for genes responsible for aromatic catabolism is a major challenge to the field of functional genomics. In particular, the evolutionary and regulatory mechanisms of aromatic catabolic pathways in the nitrogen-fixing and root-associated bacteria have been poorly documented. *P. stutzeri *A1501 was isolated from paddy soil in South China in the early 1980s for its ability to fix nitrogen under microaerobic conditions in the free-living state and to colonize rice endophytically [22-24]. As previously mentioned, aromatic compounds are highly abundant in the soil, so they can serve as a normal carbon source for A1501 when this bacterium colonizes on root surfaces of host plants. In this study, genomic analysis showed that A1501 contains sets of genes encoding enzymes and regulators involved in the biodegradation of benzoate and 4-hydroxybenzoate. Herein, we present evidence that benzoate degradation is subject to catabolite repression control. We also describe, for the first time, that low concentrations of 4-hydroxybenzoate significantly enhance the ability of A1501 to degrade benzoate.

## Results

### Genome-wide analysis of the aromatic catabolism pathways

**Figure 1:**
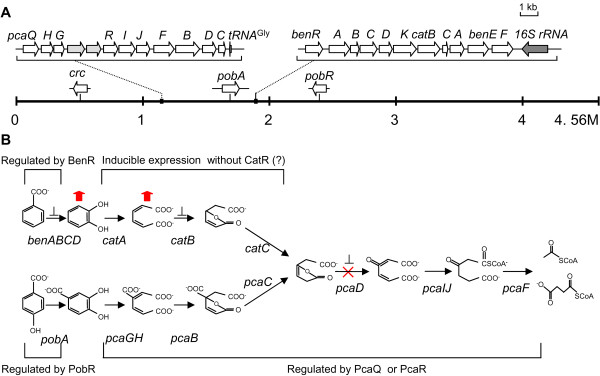
**The catechol and protocatechuate branches of the β-ketoadipate pathway and its regulation in *P. stutzeri *A1501**. (A) Localization of the gene clusters involved in degradation of benzoate and 4-hydroxybenzoate on a linear map of the chromosome. (B) Predicted biochemical steps for the catechol and protocatechuate pathways in *P. stutzeri *A1501. The question mark indicates an unknown mechanism that may be involved in the regulation of *cat *genes. Inactivation of *pcaD *is shown by "× " and accumulations of the intermediates catechol and *cis, cis*-muconate in the supernatants of the *pcaD *mutant are shown by red vertical arrows. Genes whose expression is under catabolite repression control (Crc) are indicated by "⊥".

*P. stutzeri *has recently received particular attention for its metabolic properties, including denitrification, degradation of aromatic compounds, and nitrogen fixation. Since *P. stutzeri *A1501 was originally isolated from paddy soil and because it contains sets of genes for the β-ketoadipate pathway, it should be able to utilize aromatic compounds. In our study, we observed that this strain can aerobically degrade benzoate and 4-hydroxybenzoate. As the complete genome of *P. stutzeri *A1501 was sequenced recently [20], we mapped the genes encoding the peripheral pathways for the catabolism of 4-hydroxybenzoate (*pob*) and benzoate (*ben*) in the A1501 chromosome (Figure 1A). In many soil bacteria, these peripheral pathway enzymes channel the individual substrates into one of the two branches of the β-ketoadipate pathway, namely the catechol and protocatechuate branches. Sequence comparison indicated that A1501 has genes encoding all of the enzymes involved in the two branches of the β-ketoadipate pathway. The catechol (*cat *genes) and the protocatechuate branches (*pca *genes) converge at β-ketoadipate enol-lactone. One set of enzymes, which are encoded by *pcaDIJF*, completes the conversion of β-ketoadipate enol-lactone to tricarboxylic acid cycle intermediates (Figure 1B).

**Figure 2:**
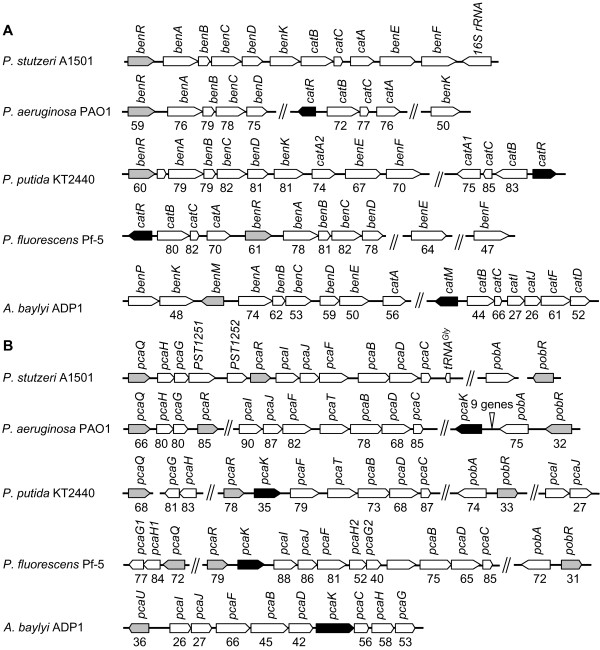
**Organization of benzoate (A) or 4-hydroxybenzoate (B) degradation gene clusters of A1501 and comparison with equivalent clusters from other bacteria**. Two vertical lines indicate that the genes are not adjacent in the genome. Numbers beneath the arrows indicate the percentage of amino acid sequence identity between the encoded gene product and the equivalent product from A1501.

In the A1501 genome, the *cat *genes are chromosomally linked with the *ben *genes and form an 11.5 kb supercluster (PST1666-PST1676). The deduced amino acid sequence of BenR in A1501 shows high similarity (61% identity) to the *P. fluorescens *Pf-5 BenR protein. However, the *catR *gene, which positively regulates the *catBC *and *catA *operons in other strains [12,25], is absent in A1501 (Figure 2A). Additionally, the *pca *genes in *P. stutzeri *A1501 are contiguous, whereas the *pca *genes are scattered over several portions of the genome in other *Pseudomonas *species, such as *P. entomophila *[21], *P. aeruginosa *[26], *P. fluorescens *[27]and *P. putida *[2] (Figure 2B). PcaR is an Icl family protein and has been reported to regulate most of the *pca *genes in the protocatechuate branch of the β-ketoadipate pathway in *P. putida *[12,28,29]. In contrast to other *Pseudomonas *strains, *pcaR *is located immediately upstream of *pcaI *in A1501 (Figure 2B). The deduced amino acid sequence of A1501 PcaR shows 85% identity to that of *P. putida *KT2440. Notably, the *pcaK *gene, which encodes a 4-hydroxybenzoate permease responsible for 4-hydroxybenzoate transport [30], is absent in A1501 (Figure 2B). The catabolic gene organization in A1501 lacks the *catR *and *pcaK *genes, a feature that is not observed in other *Pseudomonas *strains.

### Functional characterization of the β-ketoadipate pathway

**Figure 3:**
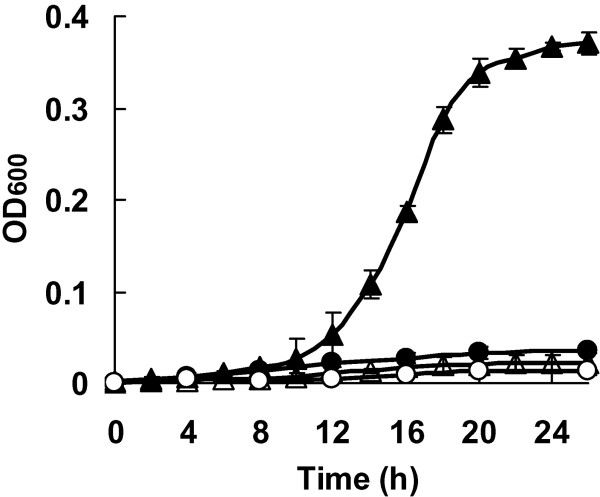
**Bacterial growth of A1501 cultured in minimal medium containing 4 mM benzoate (black triangle), 8 mM benzoate (clear triangle), 0.4 mM 4-hydroxybenzoate (black dot) or 0.8 mM 4-hydroxybenzoate (clear dot)**.

A1501 grew well on 4 mM benzoate and reached an OD_600 _of 0.5 after 24 h of incubation, whereas no growth was observed in the presence of 8 mM benzoate. A1501 grow poorly on 0.4 mM 4-hydroxybenzoate, while 4-hydroxybenzoate at concentrations above 0.8 mM completely inhibited bacterial growth (Figure 3). Further investigation of the β-ketoadipate pathway was made by constructing and characterizing three mutants: *benR *mutant A1601, *pcaR *mutant A1602 and *pcaD *mutant A1603 (Table 1). When the wild type and mutants were cultured in media containing lactate, their growth rates were not affected (data not shown). As expected, the *benR *mutant failed to grow on benzoate, and the *pcaR *and *pcaD *mutants failed to grow on 4-hydroxybenzoate as the sole carbon source. Furthermore, both the *pcaR *and *pcaD *mutants lost their ability to utilize benzoate as a carbon source. We constructed three complementary plasmids containing the entire *pcaD*, *pcaR *and *benR *genes for further growth complementation assays. Complementation of the three mutants with the corresponding complementary plasmids restored the catabolic activity, and the three corresponding complementary strains grew on benzoate as the sole carbon source (data not shown). Results from gene disruption analyses and genetic complementation tests demonstrate that the three genes are required for the growth of A1501 on benzoate.

**Figure 4:**
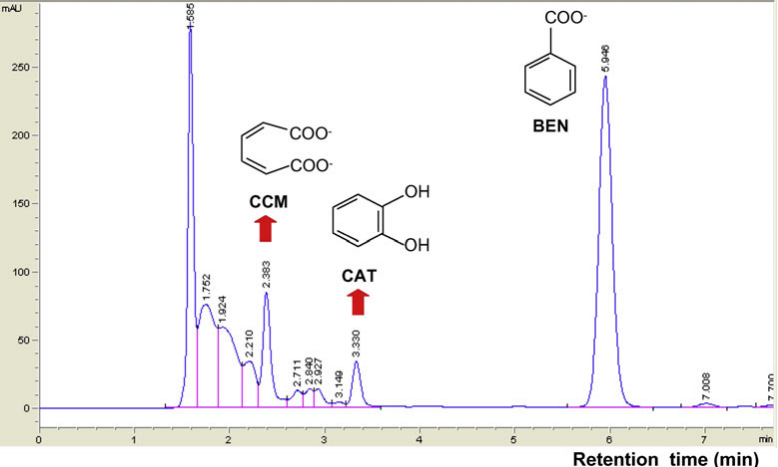
**Conversion of benzoate (BEN) to catechol (CAT) and *cis, cis*-muconate (CCM) by the *pcaD *mutant A1603**. Cells were grown for 48 h in minimal medium supplemented with 4 mM benzoate. The elution profile of compounds separated by HPLC is shown. Accumulations of the intermediates catechol and *cis, cis*-muconate are indicated by red vertical arrows.

High-performance liquid chromatography (HPLC) was used to measure the concentrations of catechol and muconate in the culture supernatants of the wild type A1501 and *pcaD *mutant A1603 grown on benzoate as the sole carbon source (Figure 4; see Additional file 1). During the initial phase of benzoate catabolism by A1501, small amounts of catechol (~30 μM) and *cis, cis*-muconate (~500 nM) were detected. After 24 h, benzoate was completely removed from the culture supernatants, and no metabolites could be detected (see Additional file 1). The inability of the *pcaD *mutant A1603 to grow on benzoate was further confirmed by HPLC analysis of culture supernatants. After 48 h, the concentration of benzoate remained almost unchanged in the culture supernatant of the mutant, while accumulation of catechol and *cis, cis*-muconate was detected by HPLC (Figure 4). As shown in Figure 1B, inactivation of PcaD completely blocked the conversion of β-ketoadipate enol-lactone to β-ketoadipate, resulting in accumulation of the intermediates catechol and *cis, cis*-muconate derived from benzoate. These results provide experimental evidence that the two branches of the β-ketoadipate pathway converge at β-ketoadipate enol-lactone and that the products of *pcaDIJF *complete the conversion of the latter to TCA cycle intermediates in *P. stutzeri *A1501, as documented in other *Pseudomonas *strains [2].

**Table 1:** Strains and plasmids used in this study

Strains or plasmids	Relevant characteristic(s)^a^	Source or reference
Strains		
*P. stutzeri*		
A1501	Wild type, Chinese culture CGMCC 0351, Ben^++^, Cat^++^, 4HBA^+^	[22]
A1601	A1501 *benR*::pK18mob Δ*benR*, Ben^-^, Cat^++^, 4HBA^+^	this study
A1602	A1501 *pcaR*::pK18mob Δ*pcaR*, Ben^-^, Cat^-^, 4HBA^-^	this study
A1603	A1501 *pcaD*::pK18mob Δ*pcaD*, Ben^-^, Cat^-^, 4HBA^-^	this study
A1608	A1601 harboring complement plasmid pLbenR	this study
A1609	A1602 harboring complement plasmid pLpcaR	this study
A1610	A1603 harboring complement plasmid pLpcaD	this study
Plasmids		
pK18mob	Km^r^; *ori*ColE1 Mob^+ ^*lacZα*^+^, used for directed insertional disruption	[45]
pRK2013	Km^r^; Tra^+^, *ori*ColE1	[46]
pLAFR3	Tcr; Tra-, Mob+, cos, RK2 replicon	[47]
pKbenR	Km^r^; 293 bp *Eco*R I-*Hin*d III fragment containing part of *benR *in pK18mob	this study
pKpcaR	Km^r^; 299 bp *Eco*R I-*Hin*d III fragment containing part of *pcaR *in pK18mob	this study
pKpcaD	Km^r^; 361 bp *Eco*R I-*Hin*d III fragment containing part of *pcaD *in pK18mob	this study
pLbenR	Tc^r^; 1041 bp *Eco*R I-*Hin*d III fragment containing *benR *with its native promoter in pLAFR3	this study
pLpcaR	Tc^r^; 1457 bp *Eco*R I-*Hin*d III fragment containing *pcaR *with its native promoter in pLAFR3	this study
pLpcaD	Tc^r^; 820 bp *Eco*R I-*Hin*d III fragment containing *pcaD *with the *lac *promoter in pLAFR3	this study

^a ^Ben^++^, good growth on benzoate; Cat^++^, good growth on catechol; 4HBA^+^, weak growth on 4-hydroxybenzoate; Ben^-^, no growth on benzoate; Cat^-^, no growth on catechol; 4HBA^-^, no growth on 4-hydroxybenzoate; Km^r^, kanamycin resistant; Tc^r^, tetracycline resistant.

**Figure 5:**
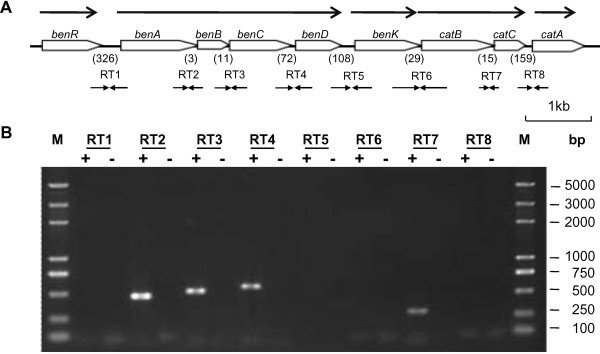
**Transcriptional organization of the chromosomal *ben-cat *region**. (A) The number of nucleotides in noncoding regions is shown in parentheses. Transcriptional units and directions are denoted by horizontal arrows in the upper panel. The designation and location of primers used for RT-PCR are in the lower panel. A pair of oligonucleotide primers is marked with a convergent arrow. (B) RT-PCR analysis of mRNA transcripts using gel electrophoresis of amplified cDNA fragments. The first and last lanes were loaded with molecular size markers. +, in the presence of inducer benzoate; -, in the absence of inducer benzoate.

As mentioned above, A1501 can grow well on benzoate, but not on 4-hydroxybenzoate, as the sole carbon and energy source. Therefore, we focused on the genetic organization of the A1501 *ben-cat *region. As shown in Figure 5A, nine *ben *and *cat *genes are in the same transcriptional orientation and the lengths of the intergenic regions vary. Gene expression was further studied by amplifying intergenic regions between adjacent genes. Eight pairs of oligonucleotide primers were designed (Table 2). As shown in Figure 5B, in the presence of benzoate, four products of their expected sizes were amplified with the PF/PR primer pairs spanning the borders of *benA-benB *(456 bp), *benB-benC *(503 bp), *benC-benD *(546 bp), and *catB-catC *(309 bp). No PCR products were observed with the PF/PR primer pairs spanning the borders of *benR-benA *(782 bp), *benD-benK *(610 bp), *benK-catB *(1074 bp), and *catC-catA *(1030 bp) in the presence or absence of benzoate. These results suggest that nine benzoate metabolic genes are organized in five transcriptional units. In particular, the *catBC *genes are co-transcribed in the presence of benzoate.

**Table 2:** Primers for RT-PCR and Quantitative Real Time RT-PCR

Primer No.	Primer name	Sequence (5'-3')	Amplified fragment^a^
1	RT1-5'	AGCGAGAACCAATGGC	782 bp *benRA *intergenic region
2	RT1-3'	TAGTCGATTCCCAGGG	
3	RT2-5'	GCACTGGATCGAGGGAGC	456 bp *benAB *intergenic region
4	RT2-3'	GTTGTGCGAGGTGCGTGT	
5	RT3-5'	GCTTTCGCTACAAGACCG	503 bp *benBC *intergenic region
6	RT3-3'	CGCACGTTGCTGATGGTC	
7	RT4-5'	CGAACCCAAACACCTCAA	546 bp *benCD *intergenic region
8	RT4-3'	CTCGGCCTCGATCTCATG	
9	RT5-5'	TACCAGGAACATGAGAT	610 bp *benDK *intergenic region
10	RT5-3'	ACGTCTACTTTTCGCATG	
11	RT6-5'	GTTCTTCTGTTGCCTG	1074 bp *benK *and *catB *intergenic region
12	RT6-3'	TCTTCGATGTCCTTAG	
13	RT7-5'	CCTTCGTCACCCTCAACA	309 bp *catBC *intergenic region
14	RT7-3'	CTTCACGCATCAGGCTCT	
15	RT8-5'	GAAGATGATCGTGAAAC	1030 bp *catCA *intergenic region
16	RT8-3'	TGAAGAAATGAATGTGC	
17	benA-5'	CGGCTCGTCCACCTATGTCTAT	186 bp internal fragment
18	benA-3'	AAACCACCGCCCTTCTTGC	
19	catB-5'	CCTTCGTCACCCTCAACAG	159 bp internal fragment
20	catB-3'	TCCAGGCTCAGGCCAAGAC	
21	pcaD-5'	TTCGCCGAGCATTTCCG	173 bp internal fragment
22	pcaD-3'	CCGATCAGTCCGCCCAT	

^a^PCR reactions were carried out with the sets of primers indicated to the left.

### BenR activates expression of the *benABCD *operon in responseto benzoate

**Figure 6:**
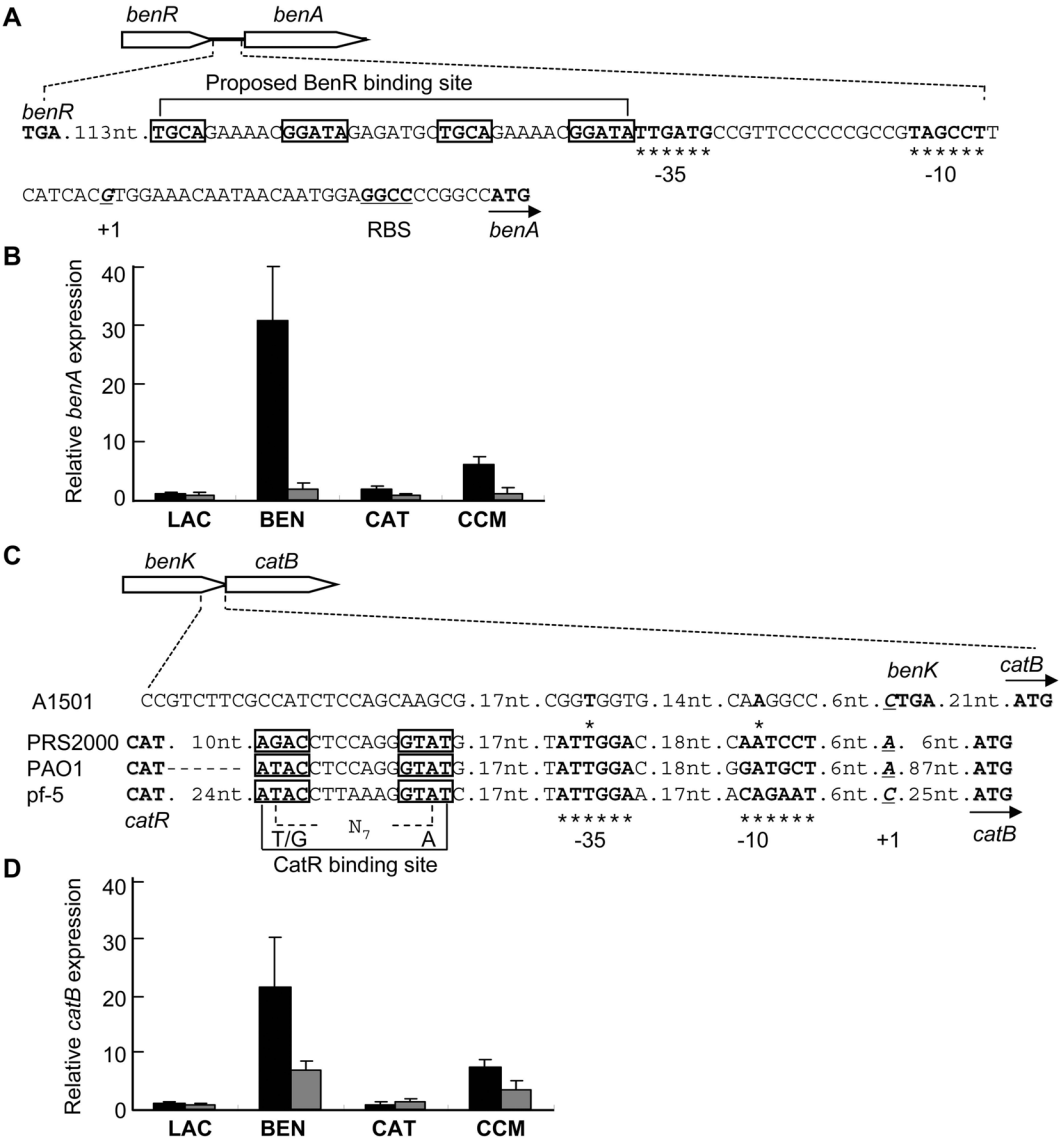
**Induction of the *benA *or *catB *promoters in culture media with several different inducers**. The putative binding site for BenR or CatR is boxed. The putative -10/-35 promoter consensus sequences are indicated by asterisks. The predicted transcriptional start site (+1) and ribosome-binding site (RBS) are underlined. (A) Nucleotide sequence of the *benR*-*benA *intergenic region of strain A1501. (B) Quantitative real-time RT-PCR analysis of relative *benA *expression level of the wild type (black bars) and *benR *mutant (gray bars) in the presence or absence of benzoate (BEN), catechol (CAT), cis, cis-muconate (CCM), and lactate (LAC). (C) Comparison of the *catB *promoter of strain A1501 with those of *P. putida *PRS2000, *P. aeruginosa *PAO1 and *P. fluorescens *pf-5. Dashes indicate gaps to obtain maximal homology. (D) Quantitative real-time RT-PCR analysis of relative *catB *expression level of the wild type (black bars) and *benR *mutant (gray bars) in the presence or absence of benzoate (BEN), catechol (CAT), cis, cis-muconate (CCM), and lactate (LAC). Relative levels of transcripts are presented as the mean values ± SD, calculated from three sets of independent experiments.

In pseudomonads, benzoate catabolism is initiated by the *benABCD *operon encoding benzoate dioxygenase (BenABC) and 2-hydro-1,2-dihydroxybenzoate dehydrogenase (BenD), whose expression is positively regulated by BenR [9,31]. In this study, we found that disruption of the *benR *gene resulted in a simultaneous loss of the ability to utilize benzoate, but this mutant could grow on catechol, suggesting that BenR might be the sole activator of expression of *benABC *in A1501. To confirm this, we searched the promoter sequence of *benA *using *in silico *analysis. The nucleotide sequence upstream of the *benABCD *operon has the following sequence features: a putative -10/-35-type promoter, a putative BenR-binding region, and a predicted translational start site (Figure 6A). Comparison with the experimentally well-characterized BenR-binding sequences in *P. putida *[9] indicated a highly conserved BenR site in the promoter region of the A1501 *benA *gene (Figure 6A). To determine whether *benR *is required for activation of the P_benA _promoter, the expression level of the *benABCD *operon was tested in the *benR *mutant A1601. Quantitative real-time PCR results demonstrated that a significant increase in transcription from the P_benA_promoter was seen in wild-type A1501 when benzoate was included in the growth medium, whereas the addition of catechol or *cis,cis*-muconate had a very weak effect (Figure 6B). When BenR was absent, transcription from the P_benA _promoter was highly repressed, irrespective of the presence or absence of the inducer (Figure 6B). As reported in *P. putida *[9], these results led us to conclude that the *benABCD *operon is under the control of BenR in response to benzoate in A1501.

### Benzoate-mediated induction of the *catBC *operon in A1501

In *P. putida*, the *catBC *operon encodes *cis,cis*-muconate lactonizing enzyme I (CatB) and muconolactone isomerase (CatC), which catalyze the second and third steps of the catechol branch of the β-ketoadipate pathway, respectively [8]. The transcription of this operon requires CatR and *cis,cis*-muconate [32]. Additionally, the translational starts of *catR *and *catBC *are separated by 136 bp of intervening DNA containing -35/-10-type promoters and the CatR-binding sites in *P. putida *[13,33]. However, we found that only 29 nucleotides are present in the noncoding regions between *benK *and *catB *in A1501, suggesting that the promoter region of the *catBC *operon overlaps with the coding region of the *benK *gene. The promoter region of the *catBC *operon from A1501 shows very low similarity to those of the three other *Pseudomonas *strains, notably the lack of the typical binding site for CatR present in the *catB *promoter region of other *Pseudomonas *strains (Figure 6C). Although a *catR *orthologue could not be identified in A1501, quantitative real-time PCR experiments indicated that benzoate has the strongest induction effect on expression of the *catBC *operon (Figure 6D). Since benzoate induces expression of *catB *in the *benR *mutant background and this mutant is unable to metabolize benzoate, we proposed that induction of the *catBC* expression is not due to the production of benzoate metabolites, such as *cis,cis*-muconate. As reported in *P. putida*, induction of the *catBC *operon requires *cis,cis*-muconate, an intermediate of benzoate degradation, and CatR, a well-studied activator in the β-ketoadipate pathway [32]. However, benzoate itself has a significant induction effect on expression of the *catBC *operon in A1501, strongly suggesting the existence of an uncharacterized regulatory mechanism.

### Benzoate degradation in A1501 is subject to carbon catabolite repression

In *Pseudomonas *and *Acinetobacter *strains, the Crc global regulator controls the expression of genes involved in benzoate degradation when other preferred carbon sources are present in the culture medium [16,17]. Based on sequence comparison, we found a Crc-like protein in the A1501 genome (Figure 1A). The A1501 Crc-like protein shows highest amino acid identity with *P. aeruginosa *Crc (86%), whereas relatively low amino acid identity (only 38%) is observed between A1501 and *A. baylyi *Crc proteins.

**Figure 7:**
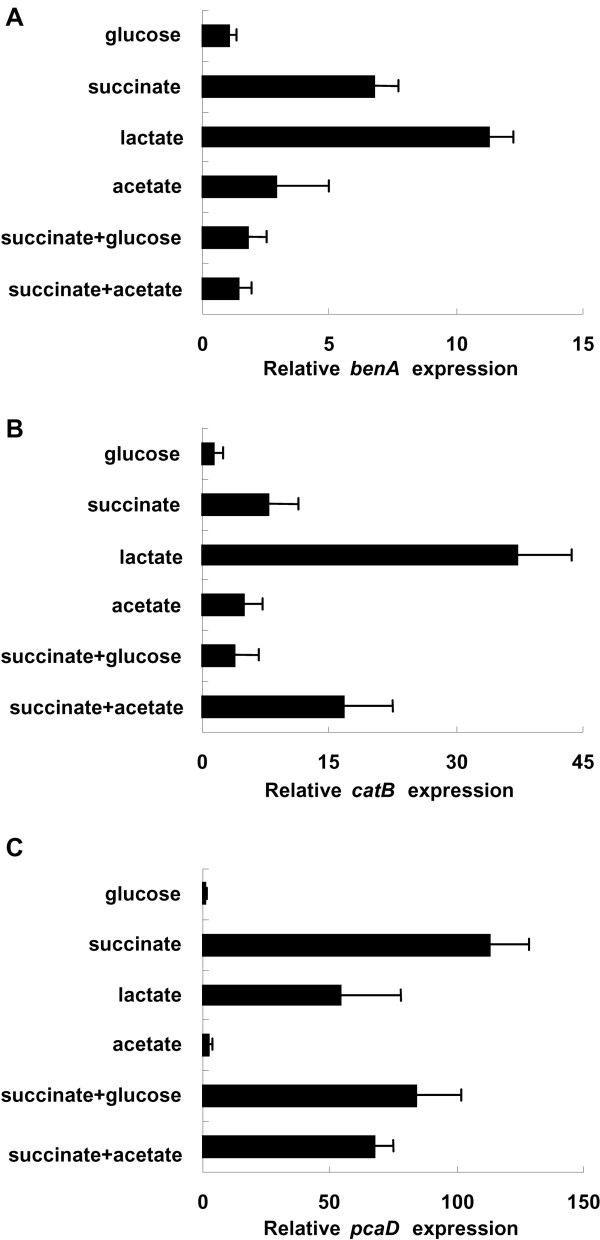
**Catabolite repression control in expression of the *benA*, *catB *or *pcaD *genes in the presence of 4 mM benzoate**. Cells were harvested and transferred into minimal medium supplemented with succinate, lactate, acetate or glucose. To induce the catabolic promoter, benzoate was added to logarithmically growing cultures. Cultures were incubated at 30°C for 2 h, and samples were collected for quantitative real-time RT-PCR analysis.

**Figure 8:**
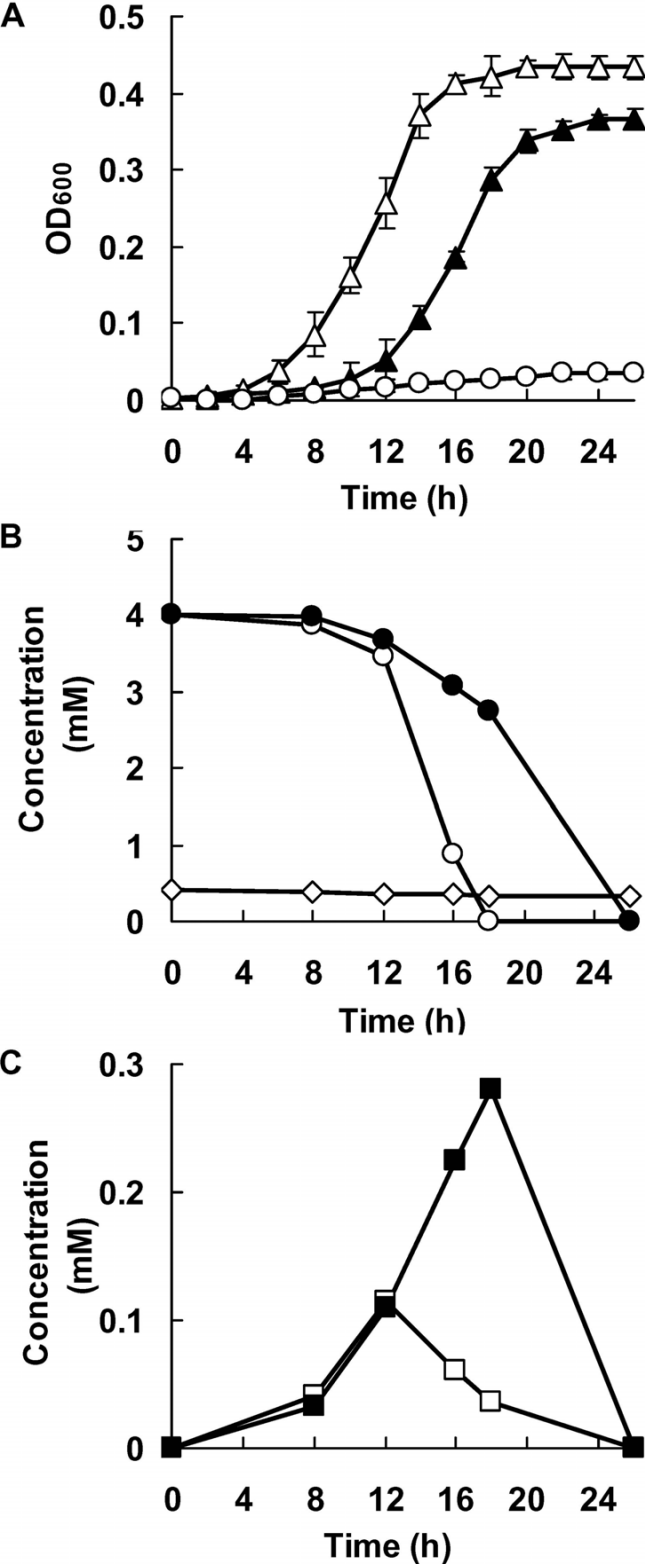
**The enhanced ability of A1501 to degrade benzoate by 4-hydroxybenzoate**. (A) Time course of bacterial growth in the presence of 4 mM benzoate (black triangle) or a mixture of 4 mM benzoate and 0.4 mM (clear triangle) or 0.8 mM (clear dot) 4-hydroxybenzoate. (B) The benzoate consumption when A1501 was cultured in minimal medium containing 4 mM benzoate (black dot) or a mixture of 4 mM benzoate and 0.4 mM 4-hydroxybenzoate (clear dot), and changes in 4-hydroxybenzoate concentrations (clear diamond) were detected by HPLC. (C) The formation of catechol derived from benzoate (black square) or a mixture of benzoate and 4-hydroxybenzoate (clear square). Samples were collected at different times and the amount of the aromatic compound in the culture supernatant was determined by HPLC.

Benzoate degradation by A1501 involves the oxidation of benzoate into catechol in a two-step process catalyzed by BenABC and BenD, two peripheral pathway enzymes of the catechol pathway. The catechol aromatic ring is converted by the action of CatA, CatB and CatC to *cis,cis*-muconate, and then to β-ketoadipate-enol-lactone, which is transformed into acetyl-CoA and succinyl-CoA by PcaD, PcaIJ, and PcaF from the β-ketoadipate pathway. Therefore, the *benA*, *catB*, and *pcaD *genes were selected for further analysis. In the presence of the inducer benzoate, highly significant differences in expression were observed, depending on the nature of the non-inducing carbon source (Figure 7). The expression of the three selected genes was most efficiently induced by benzoate when cells were grown on lactate and succinate alone, but was decreased significantly when the carbon source was glucose or acetate (Figure 8). When cells grew on lactate, the expression of *benA *and *catB *was efficiently induced by benzoate, respectively; when glucose plus succinate was used as the carbon source, induction was significantly lower (Figure 7A, B). The *benA *and *catB *genes showed a similar repression pattern to the *pcaD *gene, with the slight difference being that acetate was an intermediate-repressing carbon source. Using glucose or succinate as individual carbon sources led to a strong decreasing or increasing effect on expression of the *pcaD *gene, respectively, whereas growth on a combination of glucose plus succinate and inducer resulted in high induction (Figure 7C). These results suggest that benzoate degradation in A1501 is subject to carbon catabolite repression. Our experimental evidence, combined with the identification of the Crc-like protein in A1501, may be indicative of distinct activities of Crc at different genes or in various bacteria, as previously shown in *A. baylyi *and *P. putida *[34,35]. Further experiments are required to construct an A1501 mutant lacking the Crc-like protein and to investigate role of this protein in carbon catabolite repression.

### 4-hydroxybenzoate enhances the ability of A1501 to degrade benzoate

A study reported that high concentrations of aromatic hydrocarbons are harmful to cells because they disrupt membrane components [36]. In the plate assay, A1501 grew extremely poorly on 4-hydroxybenzoate as the sole carbon source with colonies of less than 1.0 mm in diameter after 3 days, whereas it produced normal-sized colonies (> 5 mm) on benzoate alone in the same period. These results indicate that 4-hydroxybenzoate itself directly inhibits A1501 growth, which is likely caused by the toxicity of 4-hydroxybenzoate. It is unclear whether the lack of *pcaK *results in the loss of 4-hydroxybenzoate transport, leaving A1501 unable to metabolize 4-hydroxybenzoate efficiently. In subsequent experiments, growth of A1501 was examined in a mixture of 4 mM benzoate and 0.4 mM 4-hydroxybenzoate. A1501 showed a shorter lag phase and a higher growth rate when cells were grown on the mixture than when benzoate was supplied alone (Figure 8A). Furthermore, under the latter growth conditions, the culture gradually became dark brown in color because of autoxidation of the accumulated catechol (data not shown). However, when the 4-hydroxybenzoate concentration increased to 0.8 mM, growth of A1501 was completely inhibited (Figure 8A). These results indicate that 4-hydroxybenzoate at low concentrations can enhance the ability of A1501 to grow on benzoate.

We then evaluated the effect of 4-hydroxybenzoate on the metabolism of benzoate using HPLC. When 4 mM benzoate alone was provided to the culture, it was completely consumed within 26 h, and metabolic intermediates were present. When 4 mM benzoate and 0.4 mM 4-hydroxybenzoate were provided together as growth substrates, benzoate was completely consumed within 18 h, while no discernible loss of 4-hydroxybenzoate was detected (Figure 8B). Additionally, analysis of the intracellular metabolites by HPLC revealed accumulation of catechol derived from benzoate both in the presence and absence of 4-hydroxybenzoate in the growth medium. The concentration of catechol reached 0.28 mM when A1501 grew on benzoate alone, whereas the concentration of catechol reached approximately 0.12 mM when both benzoate and 4-hydroxybenzoate were in the growth medium (Figure 8C). Collectively, these results suggest that 4-hydroxybenzoate can significantly enhance the ability of A1501 not only to degrade benzoate, but also to remove the catechol accumulated from benzoate.

## Discussion

The data presented here reveal that the sequence and organization of the *ben*, *pob*, *cat*, and *pca *genes in A1501 are very similar to those within other well-studied *Pseudomonas *strains, raising the question of whether these genes have common origins. Increasing evidence indicates that horizontal gene transfer is an efficient mechanism for introducing catabolic pathways into different bacterial genomes [37]. In general, recently acquired transferable genomic regions are associated with insertion sequence elements and mobility-related genes, whereas anciently acquired genomic regions may lose these genetic elements [38]. Furthermore, horizontally acquired DNA regions are usually chromosomally inserted in the vicinity of tRNA or rRNA genes [38]. We also discovered that an rRNA operon is located directly downstream of the *ben *gene cluster and that a tRNA-Gly gene is located downstream of the *pca *gene cluster. Although insertion sequence elements and mobility-related genes are absent, the packing of the catabolic pathway genes as well as the difference in the G+C % content from the rest of the genome favor the hypothesis that the β-ketoadipate pathway in A1501 is acquired from horizontal gene transfer, which contributes to an increased adaptability in the soil environment.

As shown in Figure 2, the gene arrangement of the *ben*, *cat*, and *pca *clusters differs between different bacteria. Apparently, various DNA rearrangements have occurred during its evolution in each particular host. Furthermore, we observed the lack of the *catR *and *pcaK *genes, a distinguishing feature of the catabolic gene organization in A1501, suggesting that gene deletion events responsible for the loss of the two genes have occurred over a long period of evolution. In most cases, the complex regulatory circuits involving the two sets of transcriptional regulators, BenR/BenM and CatR/CatM, have evolved to allow optimal expression of catabolic genes [39,40]. Unlike *P. putida *in which the transcription of the *catBC *operon requires CatR and *cis,cis*-muconate [32], we could not identify a *catR *orthologue or a consensus sequence typical of CatR-dependent promoters in A1501. In particular, benzoate, but not *cis,cis*-muconate, has a significant induction effect on the expression of the *catBC *operon in A1501. Therefore, we propose that an uncharacterized regulatory mechanism might be involved in the regulation of the β-ketoadipate pathway in A1501, but this hypothesis requires further investigation.

A1501 contains all of the enzymes involved in the 4-hydroxybenzoate degradation pathway. However, this strain shows extremely poor growth on 4-hydroxybenzoate as the sole carbon source. A plausible explanation for this observation is due to the lack of PcaK, a 4-hydroxybenzoate transporter, thereby leaving A1501 unable to metabolize 4-hydroxybenzoate efficiently. In most cases, the *pcaK *mutation had a negative effect on bacterial 4-hydroxybenzoate uptake and growth. For example, mutants blocked in 4-hydroxybenzoate transport have been identified in two biovars of *Rhizobium leguminosarum *[41]. Growth of these mutants was completely blocked when cultured on 4-hydroxybenzoate. By contrast, growth of the *P. putida pcaK *mutant was not significantly impaired on 4-hydroxybenzoate at neutral pH [30]. Furthermore, repression of 4-hydroxybenzoate transport and degradation by benzoate has been reported in *P. putida *[42]. Unexpectedly, our results indicate that low concentrations of 4-hydroxybenzoate significantly enhance the ability of A1501 to degrade benzoate, potentially due to 4-hydroxybenzoate-mediated induction of enzymes, such as PcaD, required for dissimilation of benzoate by the β-ketoadipate pathway. Pesticides and industrial wastes often contain aromatic constituents, including many that are toxic to living organisms. The degradation of aromatic compound mixtures has recently received a great deal of attention. To our knowledge, this is the first report of enhanced benzoate degradation by 4-hydroxybenzoate, highlighting its potential physiological significance. The metabolic capacity for utilizing different aromatic compounds as carbon or energy sources confers a selective advantage, notably for exposure to a mixture of aromatic compounds. The findings obtained from this study will help to investigate novel regulatory mechanisms and catabolic activities that can be of great biotechnological interest for improving the microbial degradation of aromatic environmental pollutants.

## Conclusions

We have shown that A1501 contains sets of genes encoding enzymes and regulators responsible for the entire benzoate or 4-hydroxybenzoate-degrading pathways. The unique features found in the A1501 catabolic pathway are not just rearrangements of structural genes but represent the existence of an uncharacterized regulatory mechanism and the lack of CatR, a well-studied activator in other benzoate-degrading bacteria. We also described for the first time that low concentrations of 4-hydroxybenzoate significantly enhance the ability of A1501 to degrade benzoate. More extensive studies are needed to fully understand mechanisms involved in the regulation of *cat *genes and to further improve the ability of A1501 to degrade aromatic environmental pollutants.

## Methods

### Bacterial strains, plasmids and growth conditions

The bacterial strains and plasmids used in this work are listed in Table 1. Bacterial strains were grown in Luria-Bertani (LB) and minimal lactate-containing medium (medium K), as previously described [43]. When required, carbon sources were supplemented at the following final concentrations: 4 mM glucose, 4 mM succinate, 4 mM lactate, 4 mM acetate, 4 mM benzoate, 0.4 mM catechol and 0.4 mM 4-hydroxybenzoate. The following antibiotics were added as required at the indicated final concentrations: 10 μg/ml tetracycline (Tc) and 50 μg/ml kanamycin (Km).

### Construction of nonpolar mutants

We constructed a nonpolar insertion into the *benR*, *pcaR*, and *pcaD *genes, respectively, by homologous suicide plasmid integration, as described previously [44], using pK18mob as the vector [45]. DNA fragments (~300 bp) were amplified using the total DNA of A1501 as the template and appropriate oligonucleotide primers. Oligonucleotide primers were designed to generate amplicons for the creation of nonpolar mutations enabling transcription of downstream genes. The amplicons were ligated into the vector pK18mob and the resulting plasmids were introduced into *P. stutzeri *A1501 from *Escherichia coli *JM109 by triparental conjugation using pRK2013 [46] as the helper plasmid. The nonpolar mutant strains A1601, A1602, and A1603 were generated in which *benR*, *pcaR*, and *pcaD*, respectively, were disrupted without blocking the transcription of downstream genes. Correct recombination was confirmed by PCR analysis. For further growth complementation assays, we used the broad host vector pLAFR3 to construct three complementary plasmids, pLbenR, pLpcaD and pLpcaR, as described previously [47]. Three complementary plasmids and the corresponding complementary strains are listed in Table 1.

### RT-PCR and Quantitative real-time PCR

Total RNA was isolated with an SV Total RNA Isolation System (Promega, Madison, WI, USA) and treated with RNase-free DNase I (Promega). The integrity of RNA was analyzed by agarose gel electrophoresis. To check for DNA contamination, samples were analyzed with PCR using primers for *benA*. First-strand cDNAs were synthesized from 1 μg of total RNA in a 20 μl reaction volume using the Protoscript First-Strand cDNA Synthesis Kit (New England Biolabs, Ipswich, MA, USA).

For quantitative real-time PCR (Q-PCR) experiments, primer pairs, as shown in Table 2, were designed based on the published reference genome sequence of *P. stutzeri *A1501 using the Primer 4 server. Amplicons (100 to 200 bp) and reaction specificity were confirmed by agarose gel electrophoresis and product dissociation curves. Q-PCR reactions contained 1 μl of cDNA, 10 μl of 2× QuantiTect SYBR Green PCR Master Mix (Qiagen, Hilden, Germany), 0.5 μl of each primer (20 μM stock), and 8 μl of RNase-free water. Amplifications were conducted on an ABI PRISM 7000 Real Time PCR System (Applied Biosystems, Foster City, CA, USA) under the following conditions: 10 min at 95°C, followed by 40 cycles of 15 s at 95°C, 31 s at 55°C, and 31 s at 72°C, followed by a melting-curve program (55°C to 99°C, with a 5-s hold at each temperature). Q-PCR data were analyzed using the ABI PRISM 7000 Sequence Detection System Software (Applied Biosystems). All cDNA samples were run in triplicate. The expression of l6S rRNA was used as an internal control and the signal was used to normalize variations due to different reverse transcription efficiencies. The comparative CT (threshold cycle) method was used to determine the average fold induction of mRNA by comparing the CT of the target gene to that of the reference gene, as described previously [48]. The average fold change and standard deviation from three independent RNA samples are reported for each point tested.

### High-performance liquid chromatography (HPLC) analysis

To monitor metabolism, the *pcaD *mutant and wild-type strains were grown in minimal medium supplemented with benzoate or a mixture of benzoate and 4-hydroxybenzoate. One-milliliter culture samples were centrifuged to pellet cells. Any cells remaining in the supernatant were removed by passage through a low-protein-binding, 0.22 μm pore size, syringe filter (MSI, Westborough, MA, USA). HPLC analysis was performed using an Agilent Technologies (Santa Clara, CA, USA) 1200 series chromatography system. A 20-μl sample of the filtrate was analyzed on a C18 reverse-phase HPLC column (Agilent Technologies). Elution at a rate of 0.8 ml/min was carried out with 30% acetonitrile and 0.1% phosphoric acid, and the eluant was detected at 254 nm. Under these conditions, the retention times for benzoate, catechol, *cis, cis*-muconate, and 4-hydroxybenzoate standards were 6.071, 2.388, 3.358, and 2.770 min, respectively. Peak areas corresponding to standard and experimental samples were integrated using the manufacturer's software package (Agilent Technologies).

## Authors' contributions

DL and YY carried out the experimental work, interpreted the results, and drafted the manuscript. SP, MC, and WZ constructed the nonpolar mutants. LL and WLin participated in RT-PCR and quantitative real-time PCR analysis. LG and WLiu carried out part of the HPLC analysis of intracellular metabolites. WLu and ML designed the research, analyzed data, and revised the manuscript. All authors have read and approved the final manuscript.

## Supplementary Material

Additional file 1 **Time course of benzoate consumption and metabolite formation by the wild-type strain A1501**. The elution profile of the compounds separated by HPLC is shown. Data in A-C are of samples taken at the indicated times. Conversion of benzoate (BEN) to catechol (CAT) and *cis, cis*-muconate (CCM) by A1501 is indicated by red vertical arrows. Click here for file
