# Supplementary figures and images for: Genome-wide investigation and functional characterization of the β-ketoadipate pathway in the nitrogen-fixing and root-associated bacterium Pseudomonas stutzeri A1501
Source: BMC Microbiol. 2010 Feb 8;10:36. doi: 10.1186/1471-2180-10-36 (PMC2907835; doi:10.1186/1471-2180-10-36)

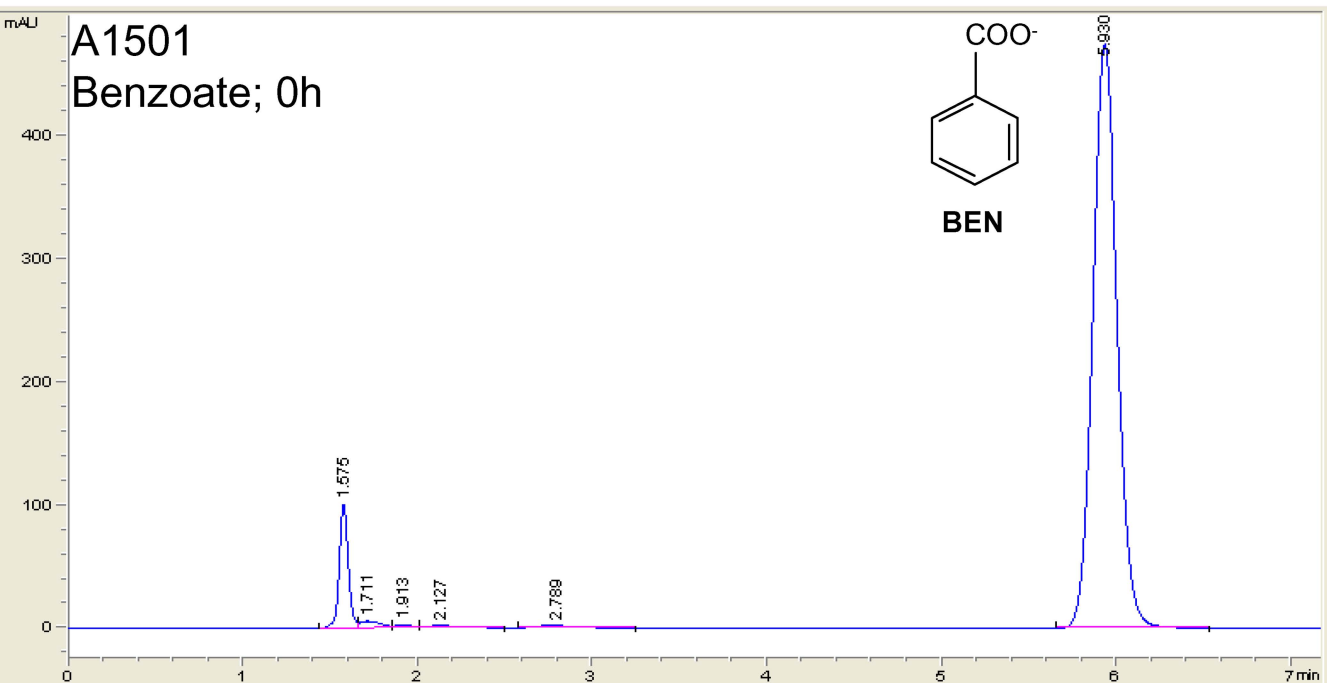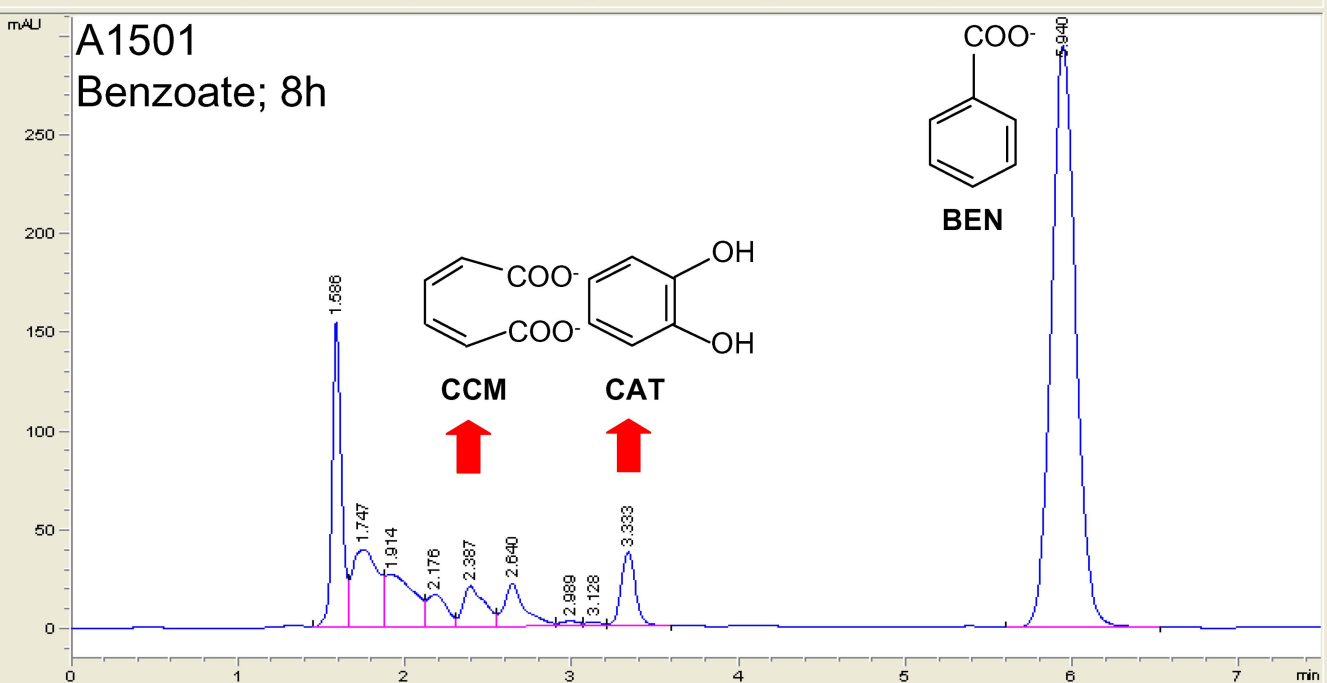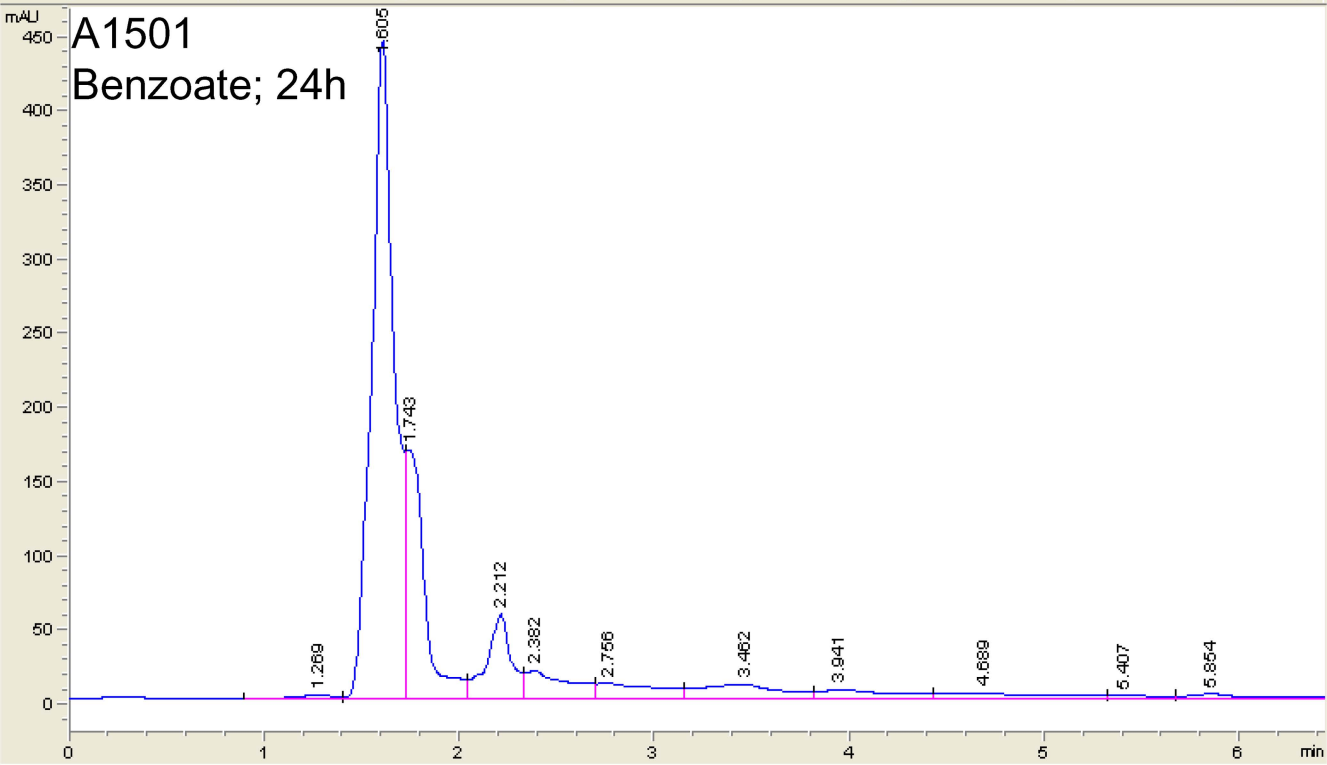

Retention time (min)

Supplement: Additional file 1 — Time course of benzoate consumption and metabolite formation by the wild-type strain A1501. The elution profile of the compounds separated by HPLC is shown. Data in A-C are of samples taken at the indicated times. Conversion of benzoate (BEN) to catechol (CAT) and cis, cis-muconate (CCM) by A1501 is indicated by red vertical arrows. [file 1471-2180-10-36-S1.PDF]
